# Structural Basis of β2 Integrin Inside—Out Activation

**DOI:** 10.3390/cells11193039

**Published:** 2022-09-28

**Authors:** Lai Wen, Qingkang Lyu, Klaus Ley, Benjamin T. Goult

**Affiliations:** 1 Department of Pharmacology, Center for Molecular and Cellular Signaling in the Cardiovascular System, Reno School of Medicine, University of Nevada, Reno, NV 89577, USA; 2 Center for Autoimmunity and Inflammation, La Jolla Institute for Immunology, La Jolla, CA 92037, USA; 3 Immunology Center of Georgia, Augusta University, Augusta, GA 30912, USA; 4 School of Biosciences, University of Kent, Canterbury CT2 7NJ, UK

**Keywords:** integrin, talin, kindlin, activation, Rap1, leukocytes, neutrophils, chemokine, structural biology, cell adhesion

## Abstract

β2 integrins are expressed on all leukocytes. Precise regulation of the β2 integrin is critical for leukocyte adhesion and trafficking. In neutrophils, β2 integrins participate in slow rolling. When activated by inside–out signaling, fully activated β2 integrins mediate rapid leukocyte arrest and adhesion. The two activation pathways, starting with selectin ligand engagement and chemokine receptor ligation, respectively, converge on phosphoinositide 3-kinase, talin-1, kindlin-3 and Rap1. Here, we focus on recent structural insights into autoinhibited talin-1 and autoinhibited trimeric kindlin-3. When activated, both talin-1 and kindlin-3 can bind the β2 cytoplasmic tail at separate but adjacent sites. We discuss possible pathways for talin-1 and kindlin-3 activation, recruitment to the plasma membrane, and their role in integrin activation. We propose new models of the final steps of integrin activation involving the complex of talin-1, kindlin-3, integrin and the plasma membrane.

## 1. Introduction

Leukocytes are essential for host defense against bacterial and fungal infection. Upon infection or sterile injury, cytokines and chemokines are released that activate the vascular endothelium and recruit circulating neutrophils. Neutrophils are often the first cell type recruited to sites of inflammation. They arrive at the site of injury by a cascade-like process involving selectin-dependent rolling followed by β2 integrin-mediated cell arrest and trans-endothelial migration [1]. This cascade requires multiple cell adhesion molecules (CAMs) and complex interplay between them to insure the appropriate response.

Neutrophil rolling is mediated by the selectin family of CAMs which orchestrate the interactions between circulating cells and the endothelium. Neutrophils express P-selectin glycoprotein ligand-1 (PSGL1, CD162) that binds all 3 selectins: P-selectin on endothelial cells and platelets, E-selectin on endothelial cells, and L-selectin on leukocytes [2]. Following attachment to the endothelial cells, the rapidly reversible interaction of selectins with their ligands on the vessel wall provides the mechanical basis of leukocyte rolling. During inflammation, chemokines such as IL-8 are released by macrophages in inflamed tissues and become immobilized on the endothelial surface [3]. The leukocyte rolling enables the cells to survey the area, and permits the interaction of leukocytes with immobilized chemokines that trigger β2 integrin activation, leading to neutrophil arrest. Transient engagement of β2 integrins with intercellular cell adhesion molecules (ICAMs) on vascular endothelium significantly reduces the rolling velocity [4]. The integrin conformation during this slow rolling is not known, but this conformation appears to require talin-1 and not kindlin-3 [5].

β2 integrins (also known as CD18) are expressed by all leukocyte types. They form a sub-class of the integrin family of CAMs. Integrins are heterodimeric adhesion receptors consisting of α- and β-subunits that are expressed on the plasma membrane. Each integrin recognizes, and connects to ICAM-1, ICAM-2 and ICAM-3 on endothelial cells and couples to the cells’ intracellular cytoskeletal and signaling machineries. There are four distinct α-subunits, αL, αM, αX and αD (or CD11a-d) that can pair with the same common β2 integrin subunit to form four distinct β2 integrin leukocyte receptors. All leukocytes express αLβ2 (CD11a/CD18), also known as lymphocyte-function-associated antigen 1, LFA-1). Myeloid cells also express αMβ2 (CD11b/CD18, also known as macrophage-1 antigen, Mac-1, or complement receptor 3, CR3). Some monocytes also express αXβ2 (CD11c/CD18, also known as complement receptor 4, CR4). Some activated lymphocytes express αDβ2. Over the years the names of these integrin receptors have changed but are standardized now as αLβ2, αMβ2, αXβ2 and αDβ2 and this nomenclature is used here. Neutrophils express mainly two of the four β2 integrins, αLβ2 and αMβ2 [6]. These two integrins have overlapping but also distinguishable roles during neutrophil arrest and migration and phagocytosis. αLβ2 is critical for neutrophil adhesion, while αMβ2 is involved in neutrophil migration, spreading, phagocytosis and activation of the respiratory burst (superoxide production). Chemokine-triggered arrest from rolling must rely on inside–out signaling, because resting integrins cannot mediate outside–in signaling. Migration, spreading, phagocytosis and activation of the respiratory burst require outside–in signaling.

Both selectin- and chemokine-induced signals converge and trigger dramatic conformational changes that result in full β2 integrin activation by inside–out signaling [7,8,9]. Integrin activation requires the recruitment and activation of the proteins talin-1 and kindlin-3 to the plasma membrane. The selectin-based signals result from neutrophil selectin, PSGL-1 engagement with the endothelial selectins, which leads to recruitment of proteins DAP12 and FcRgamma, and activation of Syk and Src kinases. Chemokine signaling starts with chemokines such as IL-8 binding to G-protein coupled receptors (GPCRs) such as CXCR1 and CXCR2. This recognition results in the separation of Gαi from the beta and gamma subunits of the GPCR, which activates phospholipase C (PLC). PLC converts the phospholipid phosphatidylinositol 4,5-bisphosphate (PIP2) to inositol trisphosphate (IP3) and diacylglycerol (DAG), leading to activation of important downstream regulators including the cascade that leads to activation of the β2 integrin. The liberation of DAG and the release of calcium from the endoplasmic reticulum in response to IP3 formation together lead to activation of a Guanine Exchange Factor (GEF) called calcium and DAG-regulated GEF1 (CalDAG-GEF1). Rap1a and b are small GTPases coordinated by CalDAG-GEF1 and are the principal effectors of the activation of integrin adhesion as they lead to recruitment of talin-1 to the plasma membrane. This recruitment can be both direct, via Rap1-talin interactions [10,11], or indirect, via intermediate proteins such as Rap1-interacting adapter molecule (RIAM) [12]. RIAM is a member of the MRL (MIG-10, RIAM, lamellipodin) family of adapter proteins [13]. Chemokine receptor activation also activates phosphatidylinositol 3-kinase (PI3K) isoforms, gamma and delta, which convert PIP2 to phosphatidylinositol 3,4,5-trisphosphate (PIP3). Since PIP3 is the preferred ligand for the pleckstrin homology (PH) domain in kindlin-3, this PIP3 production is thought to be involved in recruiting kindlin-3 to the plasma membrane.

Resting β2 integrins exist in a closed, inactive conformation, that is neither extended (E-) nor high affinity (H-). Fully activated β2 integrins are E+H+ and can bind ligand in trans (on the surface of another cell), which can cause arrest from rolling. However, between the fully inactive, E-H- and fully active, E+H+ states, two intermediate states exist, E+H- which can bind ligand in trans with low affinity and high off-rate, and E-H+ which can bind ligand in cis (on the surface of the same cell) and therefore acts as an inhibitor of adhesion [14]. Selectin ligand and chemokine receptor engagement activate intracellular signaling cascades and may result in different integrin activation states [5,15].

Integrin activation refers to conformational changes in the integrin extracellular domains, which are relayed from the binding of proteins to the integrin cytoplasmic tails. Many cytoplasmic proteins, including filamin [16], docking protein 1 [17], 14-3-3ζ [18], integrin cytoplasmic domain-associated protein 1 (ICAP-1) [19,20], α-actinin [21], talin and kindlin [22], have been reported to bind to the β-integrin cytoplasmic tail. These proteins often have overlapping binding sites on the short β-tail [23], indicating that different adaptor proteins compete for binding sites and this regulation mechanism is time- and activation-dependent. Among β-integrin binding partners, only talin and kindlin are known to be indispensable for integrin activation [24]. The phosphorylation of the α and β tails also contributes to integrin activity regulation [25]. The β2 chain is known to be phosphorylated on activation by protein kinase C [26]. α-chain phosphorylation is necessary for inducing β-chain phosphorylation in αLβ2, which may facilitate release of integrin inhibitors such as filamin and binding of integrin activators, talin and kindlin. Important phosphorylation sites in αLβ2 include S1140 on α-chain on and T758 on β-chain [27].

Although integrins mediate bi-directional signaling, we will not discuss outside–in signaling in this review. This review focuses on the final steps of β2 integrin inside–out activation. Specifically, we focus on recent advances in understanding the structures of talin-1 and kindlin-3, the only talin and kindlin isoforms expressed in leukocytes. Both proteins are essential for effective β2 integrin activation.

## 2. Talin-1 Activation

Talin-1 is a large cytoplasmic protein with a molecular weight of ~270 kDa. It contains an N-terminal head domain and a C-terminal rod domain [28]. The talin head consists of an atypical 4.1 protein, ezrin, radixin, moesin (FERM) domain. The talin FERM domain is composed of four subdomains (F0, F1, F2, and F3), and the talin rod contains thirteen consecutive helical bundles (R1-13) [28], followed by a single helical dimerization domain (DD) [29]. The talin protein is responsible for binding to a plethora of different ligands, and its 13 rod domains serve as mechanical switches [30] recruiting different ligands depending on the mechanical signals received summarized in [31,32].

In the context of integrin activation, a direct interaction between the talin FERM subdomain F3 and the cytoplasmic integrin β tail, binding to an asparagine, proline, any residue, tyrosine motif, the so-called NPxY domain (NPLF in β2) is critical for integrin activation [1,33,34]. A few amino acids preceding the membrane-proximal NPXY motif also interact with talin F3 and are necessary for talin F3 binding to the integrin NPXY motif. However, the F3 domain by itself is not a great activator and the other subdomains of the talin FERM domain are also required [35]. Talin FERM also interacts with negatively charged membrane lipids, particularly PIP2, and, as mentioned above, also binds Rap1 directly or indirectly through RIAM. The talin head is a good activator of integrins; however, maximal activation requires a mechanical linkage to the actin cytoskeleton. Indeed, there are two actin binding sites in the rod domain (ABS2 and ABS3) that form this connection [36,37] and a single connection to the actin cytoskeleton is sufficient (as a mini-talin, comprising the talin head and the ABS3 is able to activate integrins and facilitate cell spreading [38]).

**Figure 1:**
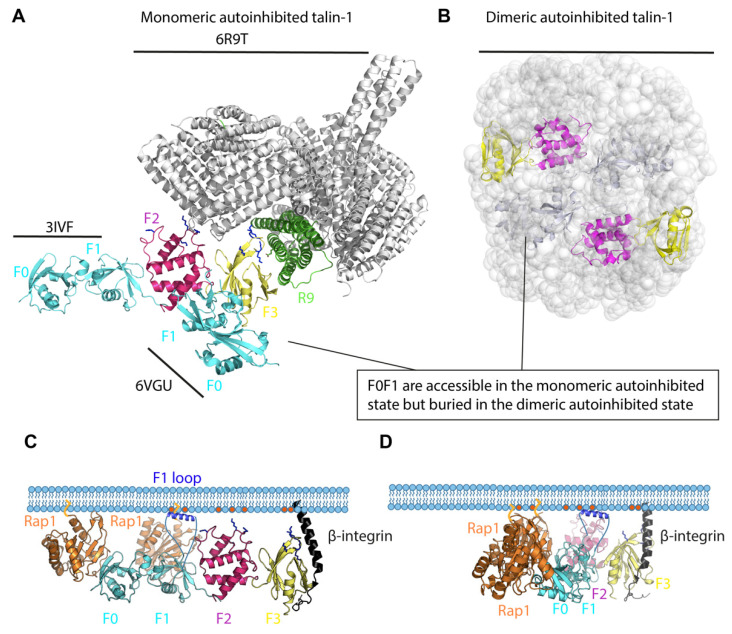
Talin autoinhibition and activation. (**A**). Monomeric autoinhibited talin-1. The F0-F1 subdomains of the linear (PDB: 3IVF) or cloverleaf (PDB: 6VGU) talin FERM domain structures are superimposed onto the monomeric cryo-EM talin structure (PDB: 6R9T, grey). The subdomain interaction between the FERM domain and the rod domain, mainly F3 (yellow)–R9 (green), is critical for autoinhibition. F0-F1 subdomains, cyan; F2, magenta. (**B**). Dimeric autoinhibited talin-1. Talin-1 forms a compact autoinhibited conformation in which the talin rods form a donut-shaped structure and the two FERM domains (F0, F1, F2, F3) are packed in parallel and buried in the donut hole. F0-F1 subdomains, cyan; F2, magenta and F3, yellow. (**C**). Integrin–talin head–Rap1–plasma membrane complex. In the linear conformation shown here, the talin F0 and F1 (cyan) interact with two Rap1 molecules (orange), which are bound to the plasma membrane through their C-terminal geranyl-geranyl moieties. The F1 loop (dark blue) and positively charged patches in F2 and F3 interact with the negatively charged PIP2 phospholipids (red) in the membrane. (**D**). A model of integrin–talin head–Rap1–plasma membrane complex based on the cloverleaf talin-1 FERM domain. It is not known whether the linear and cloverleaf conformations exist exclusively, in parallel, or sequentially (see text for discussion).

Many proteins are regulated by autoinhibition [39] and talin is no exception, adopting autoinhibited closed conformations [40] mediated by the integrin-binding F3 subdomain binding to the R9 rod domain [41,42]. This autoinhibited talin is predominantly cytosolic in resting cells, and structural insight on this autoinhibition has been revealing. The high-resolution cryo-electron microscopy (cryoEM) structure of a monomeric autoinhibited form of talin-1 was recently solved [43] and is shown in Figure 1A, based on pdb structures 6R9T [43], 3IVF [44] and 6VGU [45]. Talin-1 expressed recombinantly in bacteria is monomeric [43] although the talin-1 extracted from turkey gizzards is dimeric [46]. It is likely that talin can adopt multiple different activation states, a theme that runs through this whole review. Electron microscopy (EM) studies of full-length talin-1 extracted from turkey gizzard indicate that talin-1 forms a compact autoinhibited dimer (Figure 1B) [46]. In the autoinhibited monomeric talin (Figure 1A), the N-terminal F0 and F1 domains are not resolved in the cryo-EM structure of talin-1 [43]. This suggests that the F0F1 double domain module is flexible in autoinhibited monomeric talin. However, structures of the talin FERM domain exist (PDB 3IVF and 6VGU) and in Figure 1A, the F0F1 domains were added in the two different conformations from the separate crystal structures. In both conformations, the F0 and F1 domains are accessible for binding to membrane-bound Rap1, but the F3 domain, which can bind the membrane-proximal NPLF motif in β2, is buried in the globular talin-1 and masked by the R9 subdomain of the talin rod (Figure 1A). The PIP2 binding sites in the F2 and F3 domains are also masked (Figure 1A). In contrast in the structure-based modelling of the dimeric autoinhibited state, the F0 and F1 domains are buried inside the structure and thus not available for Rap1 binding. Similarly, the F2 and F3 domains are also buried, so integrin and membrane phospholipid binding are also occluded (Figure 1B). Clearly there must be activation steps that need to occur that prime the talin molecule for integrin binding and activation.

### The Talin Head—A Rigid Clover-Leaf or a Versatile Modulatable Structure?

The structures of FERM domains are defined by a classical clover leaf arrangement whereby the F1, F2 and F3 domains interact with each other. However, the multiple X-ray structures of the talin head domain have hinted at a plasticity of the talin head that makes it unique in the FERM domain family. In this section we briefly review the structure of the talin head as it impacts on the models of the regulation of integrin activation complex.

In all structures of the talin head to date, including the structures of F0F1 and F2F3 double domain modules, the orientation of F0 relative to F1 and F2 relative to F3 is fixed. That means the F0F1 and F2F3 double domains have fixed orientations and behave as single units. The thing that varies between the structures is the orientation of the F0F1 module relative to the F2F3 module. In the full-length talin cryoEM structure, the F0F1 module is not resolved, indicating that it is flexible relative to F2F3.

**Figure 2:**
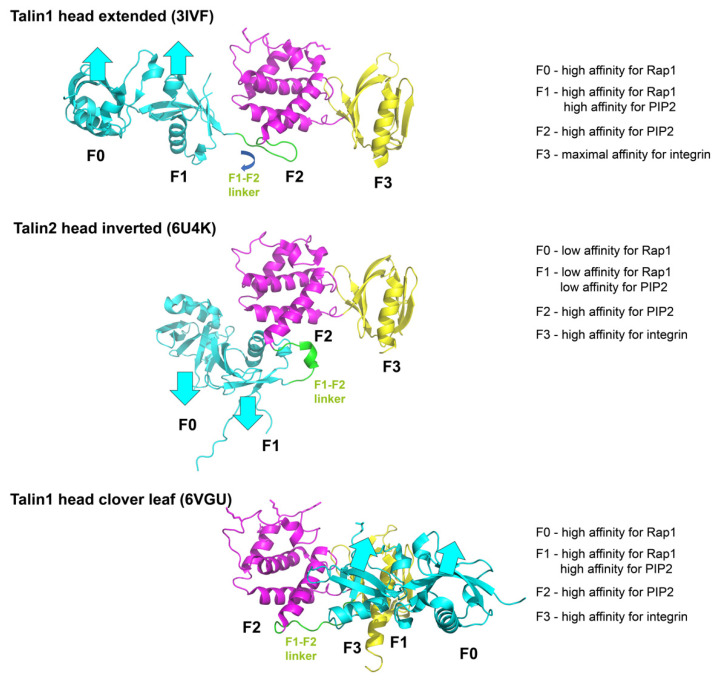
A model of dynamic regulation of integrin activation states by talin head. Upper panel: Talin-1 head extended conformation. The F0F1 subdomains (cyan) facing the plasma membrane have high affinity for Rap1. F1 and F2 (magenta) have high affinity for PIP2. F3 (yellow) has maximal affinity for integrin. Middle panel: Talin-2 head inverted conformation. The F0F1 subdomains are inverted, away from the membrane, with low affinity for Rap1 and PIP2. Lower panel: Talin-1 head cloverleaf conformation. The F0F1 subdomains facing the plasma membrane have affinity for Rap1 and PIP2; F3 has high affinity for integrin.

Crystallographic analysis of the truncated talin head domain (F0, F1, F2, F3) alone revealed that talin-1 head (with truncation of the F1 loop) can adopt a linear conformation [pdb 3IVF and 4F7G [44,47]] (Figure 2 top). However, a more recent crystal structure of the full length talin head with the F1 loop as well as a C-terminal polylysine motif used a clever chimera-strategy, whereby appending the β3 integrin tail to the start of F0 enabled integrin binding to the F3 domain and stabilized the closed conformation. This approach showed that talin-1 head can fold into a classical FERM domain clover-leaf structure [pdb 6VGU [45]] (Figure 2 bottom). The structure of the intact talin-2 FERM domain revealed a conformation that was between these two states [pdb 6U4K [48]] and provides strong evidence in support of the F1-F2 linker being a site of talin regulation (Figure 2 middle). Here, the F0F1 are inverted 180° to the F2F3 relative to their orientation in the linear structure.

These findings suggest two models: either the clover-leaf structure is the correct structure and the others are artefacts, or the talin head has a unique propensity to adopt different conformations in response to different signals. The latter might allow different signaling pathways to modify the talin head conformation, thus integrating signaling pathways into the integrin activation process. The second model is more likely, supported by experimental evidence. As mentioned above, the talin FERM domain is atypical. The talin FERM domain is the only FERM domain from which the subdomains can be readily purified in isolation. By contrast, the kindlin, MyosinX and IDOL FERM domains cannot be subdivided because individual subdomains are insoluble. Third, the fact that F0F1-F2F3 has been crystalized in two conformations supports the notion of flexibility. Thus, we posit that the F1-F2 linker is a regulatory feature of the talin head. In particular, the inversion is of interest and might indicate a novel regulatory mechanism. There are significant spatial constraints, because the known ligands for the talin FERM domain are all membrane bound (Rap1, PIP2) or transmembrane (integrin β). If talin is bound to the membrane with the F0 and F1 aligned so that they are in an orientation to bind Rap1, then we predict the affinity for Rap1 will be high. However, if F0F1 is inverted its affinity for Rap1 will be markedly lower (Figure 2).

The talin F0F1 domains are required for robust activation [35]. Both F0 and F1 bind to Rap1 via their Ras Binding Domain (RBD) fold [10], and F1 also can bind the membrane, but only when the membrane is negatively charged [49]. This leads to a model whereby the F0F1 together might provide a sophisticated mechanism for coincidence detection of the signaling pathways, and a way that two signals can synergistically enhance adhesion. If Rap1 is not active, or if the PIP2 generation is low, then the tripartite interaction between F0F1, 2xRap1 and the membrane PIP2 will not form. So, depletion of PIP2, or low Rap1 activity, will prevent the adhesion complex from being stabilized. Furthermore, the F2F3 interaction with the membrane and integrin provides an intermediate capacity for integrin activation [35]. Thus, this mechanism may limit adhesion prior to receiving these multiple input signals (active Rap1 and PIP2 colocalized). This conformation exposes the talin-binding sites for β2, PIP2 and Rap1, each necessary for integrin activation (Figure 1C). The stoichiometry could be 2:1:1, where each of the 2 Rap1 molecules binds F0 and F1, respectively, and each talin head domain binds one β2 cytoplasmic tail through its F3 domain. Membrane localization is facilitated by the F1 loop (highlighted in blue in Figure 1C) that binds PIP2 (red dots in Figure 1C).

It seems that the F1 loop, and the charged residues in F2 and F3 are also close enough to the plasma membrane to bind the negatively charged PIP2 phospholipids. When bound by talin, the β-integrin tail switches from a 25-degree tilt angle [50] and becomes oriented perpendicular to the plasma membrane (Figure 1C). The two possible conformations of the talin head domain (Figure 1C,D) will be discussed in more detail below.

Two pathways lead to talin-1 activation and Rap1 plays a central role in both, (1) direct activation of talin-1 by Rap1 through direct binding of Rap1 to talin-1 (Rap1 to talin-1 F0 and F1), and (2) indirect activation of talin-1 by Rap1 mediated by RIAM (Rap1-RIAM-talin-1 axis). Rap1 is targeted to the membrane by covalent geranyl-geranylation of a cysteine residue [51]. Upon activation, Rap1 recruits RIAM, which provides an anchor for talin-1. RIAM binds to the F3 domain of the talin head and the R2, 3, 8 and 11 domains in the talin rod [46,52]. This multisite binding could be involved in the unfolding of autoinhibited talin. Interestingly, RIAM is absent in platelets and important in only some leukocytes [53].

NMR studies suggested direct but low-affinity interaction between Rap1b and the talin-1 F0 domain [49]. However, the direct interaction of talin with Rap1 is important for adhesion of Dictyostelium cells [54]. A mutation in the F0 domain blocking the Rap1 binding leads to embryonic lethality in flies [55]. Similar mutations blocking the Rap1 binding to talin-1 F0 domain (R35E or K15A, R30A, R35A-Tln1^3mut^) had only mild effects on platelet integrin activation in mice [56,57]. Later work showed that a second Rap1 binding site (R118) in the F1 domain is also involved in integrin activation [10]. The R35E, R118E double mutant failed to activate integrin. The importance of the two pathways varies between cell types as revealed by mouse data [53]. The Rap1-talin1 interaction appears to be a general mechanism as it is ubiquitously present in different cell types (platelets, leukocytes including effector or regulatory T cells), and conserved in different organisms such as Dictyostelium, flies and mice. The Rap1-RIAM-talin-1 pathway is required for integrin activation in neutrophils and effector T cells.

Full activation of talin-1 needs the cooperation of Rap1 binding and PIP2-mediated binding of talin-1 to the plasma membrane (Figure 1C) [10,47,58]. The PIP2 binding sites are in positively charged patches in the talin-1 F2 and F3 domains [59,60,61]. The F1 loop also contains positively charged amino acids (R146, R153, K156) that are effective in binding to membrane lipids [46]. The binding of talin-1 to membrane PIP2 appears to stabilize the weak interactions of talin-1 with the membrane-bound Rap1 and the β2 cytoplasmic domain.

## 3. Kindlin-3 Activation

**Figure 3:**
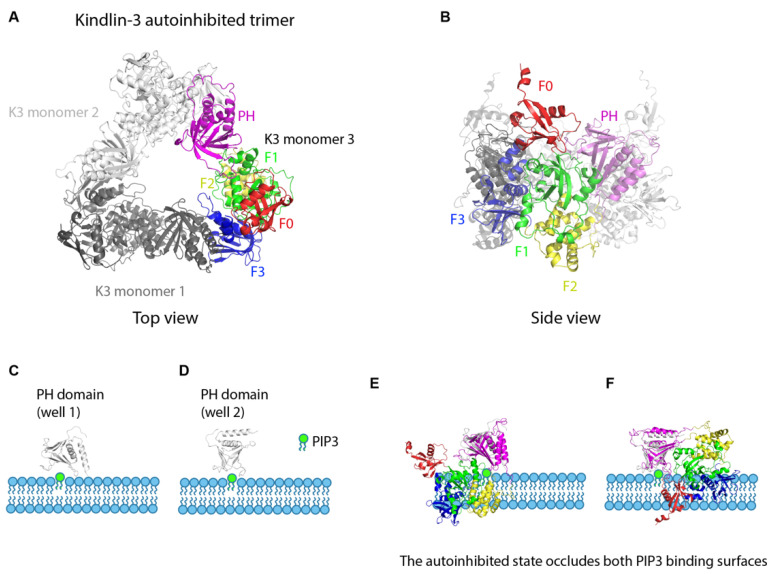
Kindlin autoinhibition and activation. (**A**). Crystal structure of the kindlin-3 autoinhibited homotrimer (top view). Kindlin-3 PH domain (magenta) interacts with the F3 subdomain of another kindlin-3 molecule (grey), thereby masking the integrin binding site of kindlin-3. F0, red, F1, green, F2, yellow, F3, dark blue. (**B**). Crystal structure of the kindlin-3 autoinhibited homotrimer (side view). (**C**,**D**). Two wells [well 1 (**C**) and well 2 (**D**)] of the free kindlin-3 PH domain binding to PIP3 (green) in the plasma membrane. (**E**,**F**). The autoinhibited state occludes both PIP3 binding surfaces of the PH domain. Superimposition of the autoinhibited kindlin-3 monomer onto the kindlin-3 PH domain in (**C**,**D**) shows that at least 2 domains would clash with the membrane containing PIP3.

Kindlin-3 is known to be required for β2 integrin activation [62,63,64]. The mechanisms of how integrin activation is regulated by kindlin-3 are not well understood. Like talin-1, kindlin-3 is also a FERM domain-containing cytoplasmic protein with 4 subdomains (F0, F1, F2 and F3) [65]. The F1 domain contains a long flexible loop containing a poly-lysine stretch in the F1 domain [66] and the F2 subdomain has an inserted PH domain. Crystallographic analysis of recombinant kindlin-2 and kindlin-3 with the PH domain and the F1 loop truncation showed the classical clover-leaf conformation of FERM domains [67,68]. Kindlin-2 homodimerization was first observed in a kindlin-2 lacking the PH domain and F1 loop [67]. The dimer formation is mediated by F2-F2 domain interaction and has very slow kinetics [67]. Whether and how kindlin dimerization occurs in cells remains elusive. The PH domain alone was also crystalized at high resolution [69]. Crystallization of full length kindlin-3 revealed that kindlin-3 forms a homotrimer [70]. The F3 domain of kindlin-3 is occluded by the PH domain of a neighboring kindlin-3 protomer (Figure 3A,B). Because the kindlin-3 F3 domain binds the PH domain in homotrimeric kindlin-3, this conformation cannot bind the integrin β chain. The free kindlin PH domain can bind PIP3 in the membrane with 2 different energy minima (“wells”) (Figure 3C,D). However, the autoinhibited homotrimeric kindlin-3 cannot bind PIP3 as both PIP3 binding wells are occluded; i.e., would clash with the membrane (Figure 3E,F).

The kindlin-3 PH domain is necessary for recruitment of kindlin-3 to the plasma membrane [71,72]. To trigger kindlin-3 recruitment to the plasma membrane, the auto-inhibited kindlin-3 homotrimer must be broken up. The mechanism of this dissociation is not known. The monomeric kindlin-3 would be expected to bind PIP3 (through its PH domain) and integrin β (through its F3 domain).

## 4. Talin-1 and Kindlin-3 Binding to β2 Integrin

**Figure 4:**
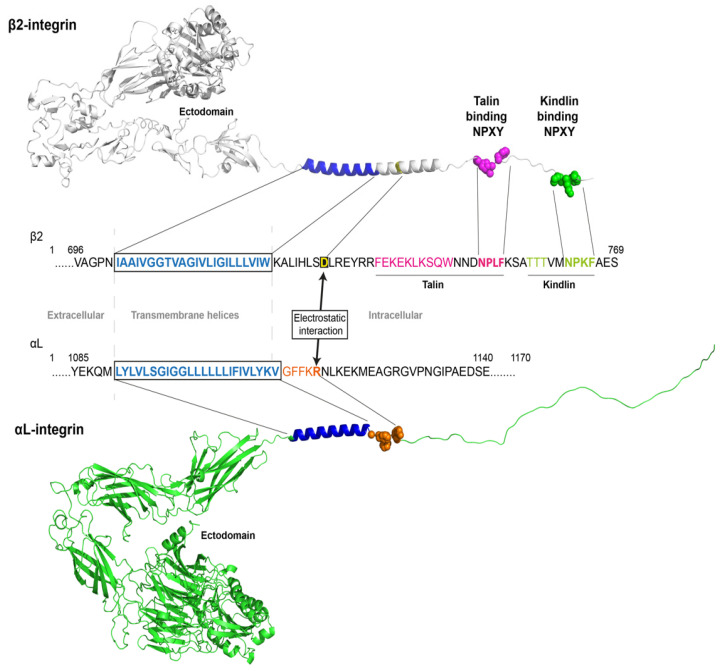
Integrin structure and binding sites for talin and kindlin. The integrin αLβ2 consists of an ectodomain followed by transmembrane helices and intracellular domains, respectively. Talin-1 and kindlin-3 bind to distinct binding sites (NPLF, membrane-proximal and NPKF, membrane-distal) on the β2 subunit, respectively. The inhibitory salt bridge (electrostatic interaction) of the intermembrane clasp is formed between the αL-arginine in the highlighted GFFKR sequence (red) and β2-aspartic acid (boxed in black).

Both talin-1 and kindlin-3 bind to the integrin β cytoplasmic domain, which contains two NPXY sites. The membrane-proximal NPXY site (sequence NPLF in β2) is the primary talin-binding site (Figure 4). The membrane-distal NPXY site (sequence NPKF in β2) is the primary kindlin binding site. Biochemical analysis suggests that talin and kindlin can bind to integrin β independently, without blocking each other’s binding [73,74] enabling both to engage simultaneously. The Ser/Thr-rich intervening sequence (sequence TTT in β2) before the membrane-distal NPXY sites has also been reported to bind to kindlin-3 and is essential for β2-mediated cell migration [75].

As mentioned above, talin and kindlin autoinhibition provides a plausible mechanism for maintaining integrin in a low-activation state. In this E-H- state, the transmembrane helices (blue in Figure 4) of β2 and the α chain (αL in Figure 4) are held together by multiple mechanisms. Here, we highlight the inner membrane clasp, which mainly operates by electrostatic interaction between the membrane-proximal R in the αL GFFKR sequence (red in Figure 4) and the membrane-proximal D in β2 (black box in Figure 4). The β2 and αL ectodomains are shown in the bent-closed conformations based on pdb structures.

## 5. A Model of Integrin Activation

**Figure 5:**
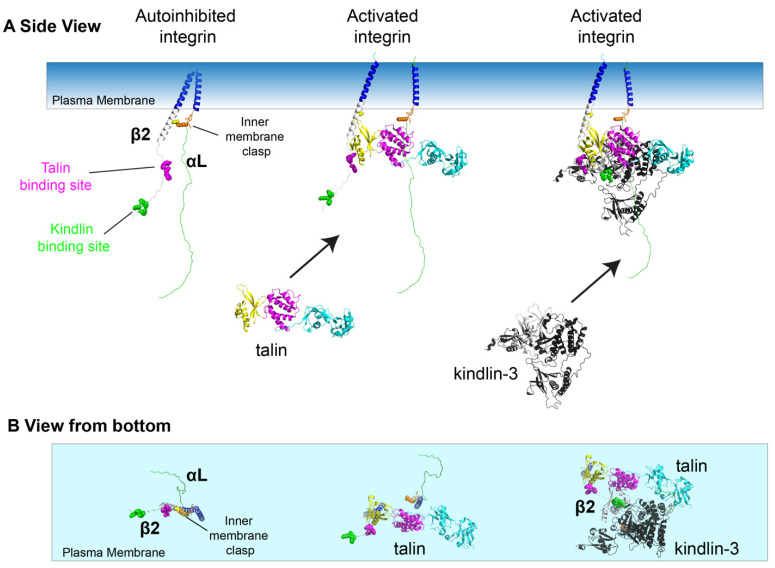
Talin and kindlin interactions to activate integrin. (**A**). Side view. Autoinhibited integrin is maintained by the inner membrane clasp (left panel, orange). Talin-binding site in magenta, kindlin binding site in green. Talin binding to the membrane proximal NPLF leads to breaking of the inner membrane clasp and αLβ2 leg separation (middle panel). Kindlin-3 (black) can bind the β2 tail simultaneously or sequentially with talin-1. Only the open conformation of the talin head is shown here, as the F1 domain moving out of the way creates space for the kindlin-3 to dock in more easily to form an activated integrin-talin-kindlin complex. (**B**). Bottom view of cooperation of talin-1 and kindlin-3 in integrin activation corresponding to the side views shown in panel (**A**).

In its resting state, integrins are bent (not extended, E-) and low affinity (not high affinity, H-) as shown in Figure 4. In that state, the integrin α and β chains are close to each other, held together by the inner membrane clasp (Figure 5A, left panel, orange) and other mechanisms (not shown). When the talin head domain binds integrin β through the membrane-proximal NPXY motif, the inner membrane clasp is broken and the α and β transmembrane helices move apart (Figure 5A, middle panel). Kindlin-3 has also been implied in releasing the inner membrane clasp [76]. Kindlin-3 can bind the membrane-distal NPXY motif (Figure 5A, right panel), even when talin-1 is already bound [73,74]. It is not known whether talin or kindlin binds first in inside–out activation. In Chinese hamster ovary (CHO) cells expressing β1 integrin, Horwitz and colleagues observed that kindlin enters nascent focal adhesions before talin [77]. This finding suggests that the three panels of Figure 5A should not be interpreted as a time sequence. Figure 5B shows the bottom view corresponding to the side views in Figure 5A. The right panel illustrates that the talin head domain and kindlin-3 can bind β2 integrin without interfering with each other.

The talin head domain (F0F1, cyan, F2, magenta, F3, yellow) has been crystallized three times, yielding different structures. The talin1 head extended conformation (PDB 3IVF, Figure 2, top) has high affinity for integrin, PIP2 and Rap1. Since Rap1 binding is known to be required for integrin activation by talin, this structure is possibly the functionally fully active structure. Talin2 head has also been reported to exist in a “head inverted” conformation (PDB 6U4K, Figure 2 middle), which has lower affinity for Rap1 and PIP2 than the cloverleaf conformation (Figure 2, bottom). The cloverleaf conformation reported in PDB 6VGU has high affinity for integrin β, and is compatible with interactions with Rap1. It is possible, but not proven that the head-inverted structure may be intermediate between the cloverleaf and the head extended structure.

## 6. Integrating the Known Steps of Integrin Activation

**Figure 6:**
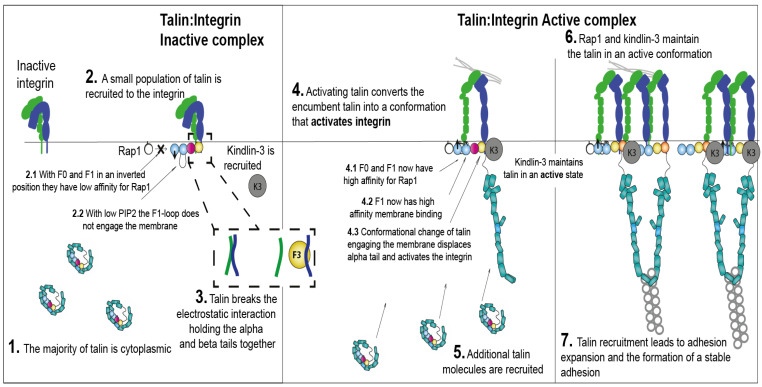
A model of integrin activation by talin-kindlin-Rap1. (1). The majority of talin is in the cell cytoplasm. Integrin adopts a conformation with bent ectodomain and low affinity for ligands. (2). Integrin activation is initiated by a small population of talin being recruited to the integrin. Talin adopts a conformation with inverted F0-F1, in which F0 has low affinity for the membrane-bound Rap1 and F1-loop has low affinity for PIP2. (3). Talin F3 binding to integrin β leads to breaking of the inner membrane clasp. (4). The F0F1 subdomains are inverted, allowing for F0 and F1 to engage Rap1 and PIP2. Displacing the α tail leads to separation of the integrin α and β transmembrane domains and conversion of integrin into the active conformation (extended high affinity). Kindlin-3 cooperates with talin and maintains talin-integrin in an active state. (5). More PIP2 and active Rap1 are generated at the plasma membrane, which recruits additional talin molecules to form the Rap1-talin-integrin-membrane complex. (6). The active integrin conformation is maintained by a complex containing Rap1, talin-1, kindlin-3 and integrins. This complex is linked to the actin cytoskeleton through talin. Kindlin-3 may also contribute to the clustering of integrins at this stage. (7). Talin recruitment and integrin activation engaging ligands leads to more molecule recruitment, resulting in adhesion expansion, strengthening and stabilization.

Given all the known structures and interactions, how might integrin actually be activated in a cell? Figure 6 lays out a hypothesis of how this might happen that is consistent with the known binding events. Initially, integrin is bent (E-H-) and most talin is cytoplasmic (Figure 6: 1), as a monomer or dimer. Chemokine receptor activation is known to produce patches of PIP2 and PIP3 in the inner leaflet of the membrane. The exposed PIP2 may be sufficient to initiate some talin recruitment, which may be sufficient to break the inner membrane clasp (Figure 6: 2). This would result in a separation of the integrin α and β transmembrane domains (Figure 6: 3). There is experimental evidence that leg separation is associated with integrin activation ([78], Figure 6: 4), but the mechanistic details are unknown. At this stage, additional talin may be recruited to the membrane (Figure 6: 5). It is unknown how the E+H-, E-H+ and E+H+ integrin conformations are achieved and stabilized. Rap1, talin-1 and kindlin-3 are thought to maintain the integrin in a fully active conformation (E+H+, Figure 6: 6). Since talin has actin binding sites in the rod domain, now active integrin may be organized by actin binding through talin (Figure 6: 7). Adhesion complexes rapidly grow, as autoinhibition of integrin, talin-1 and kindlin-3 are relieved. Further molecules are recruited, assemble and are stabilized at the adhesion sites, possibly making this an autocatalytic process. Some of the steps proposed in Figure 6 are currently just conjectures and remain to be tested experimentally.

## 7. Conclusions

We lay out a model for integrin activation by talin and kindlin binding. Both talin-1 and kindlin-3 are normally autoinhibited. How this autoinhibition is relieved is not known, but it is possible that formation of PIP2 and PIP3 patches upon chemokine receptor activation may be sufficient to initiate the process. Both talin-1 and kindlin-3 become active when recruited to the plasma membrane. Active Rap1 plays a central role and so the convergence of Rap1 and PIP2 provides a nexus at which talin-1 and kindlin-3 can help recruit and activate, or stabilize the integrin-activating form of talin. Both talin-1 and kindlin-3 can bind distinct domains in β2. In the absence of talin-1 or kindlin-3, β2 integrins cannot be activated. Talin-1 or kindlin-3 binding can release the inner membrane clasp and thus promote integrin leg separation and activation. Sophisticated mechanisms orchestrate the interactions between talin, kindlin, Rap1 and integrin, ensuring that the appropriate adhesion response occurs only in response to the correct signals.

## Data Availability

Not applicable.
